# Crowdsourcing a Normative Natural Language Dataset: A Comparison of Amazon Mechanical Turk and In-Lab Data Collection

**DOI:** 10.2196/jmir.2620

**Published:** 2013-05-20

**Authors:** Daniel R Saunders, Peter J Bex, Russell L Woods

**Affiliations:** ^1^Schepens Eye Research InstituteBoston, MAUnited States; ^2^Schepens Eye Research Institute, Massachusetts Eye and EarBoston, MAUnited States

**Keywords:** Internet, web, crowdsourcing, free recall

## Abstract

**Background:**

Crowdsourcing has become a valuable method for collecting medical research data. This approach, recruiting through open calls on the Web, is particularly useful for assembling large normative datasets. However, it is not known how natural language datasets collected over the Web differ from those collected under controlled laboratory conditions.

**Objective:**

To compare the natural language responses obtained from a crowdsourced sample of participants with responses collected in a conventional laboratory setting from participants recruited according to specific age and gender criteria.

**Methods:**

We collected natural language descriptions of 200 half-minute movie clips, from Amazon Mechanical Turk workers (crowdsourced) and 60 participants recruited from the community (lab-sourced). Crowdsourced participants responded to as many clips as they wanted and typed their responses, whereas lab-sourced participants gave spoken responses to 40 clips, and their responses were transcribed. The content of the responses was evaluated using a take-one-out procedure, which compared responses to other responses to the same clip and to other clips, with a comparison of the average number of shared words.

**Results:**

In contrast to the 13 months of recruiting that was required to collect normative data from 60 lab-sourced participants (with specific demographic characteristics), only 34 days were needed to collect normative data from 99 crowdsourced participants (contributing a median of 22 responses). The majority of crowdsourced workers were female, and the median age was 35 years, lower than the lab-sourced median of 62 years but similar to the median age of the US population. The responses contributed by the crowdsourced participants were longer on average, that is, 33 words compared to 28 words (*P*<.001), and they used a less varied vocabulary. However, there was strong similarity in the words used to describe a particular clip between the two datasets, as a cross-dataset count of shared words showed (*P*<.001). Within both datasets, responses contained substantial relevant content, with more words in common with responses to the same clip than to other clips (*P*<.001). There was evidence that responses from female and older crowdsourced participants had more shared words (*P*=.004 and .01 respectively), whereas younger participants had higher numbers of shared words in the lab-sourced population (*P*=.01).

**Conclusions:**

Crowdsourcing is an effective approach to quickly and economically collect a large reliable dataset of normative natural language responses.

## Introduction

Internet-based crowdsourcing of medical studies has had a number of successes in recent years [1]. Using open calls on the Web, researchers have been able to recruit large, and sometimes specialized, populations to contribute data, with low expenditure of resources. For example, 20,000 members of the 23andMe genome-sequencing community responded to a detailed survey about their phenotype [2], over 500 individuals with developmental prosopagnosia were identified through self-testing on the research group’s website [3], and thousands of online participants contributed information about their off-label drug use [4]. Traditional recruiting and testing to collect these datasets would have been expensive—in some cases prohibitively so—whereas in these cases, data were contributed freely.

In most uses of crowdsourcing in medical research to date, the primary data consist of categorical responses. However, for many purposes it would be valuable to quickly and inexpensively collect large natural language datasets in response to an open-ended question or prompt. Such a process could be used to norm projective psychological tests or to compile qualitative descriptions of disease symptoms or commonly experienced side effects of treatment. In one recent application of crowdsourcing, workers gave qualitative, free-text feedback on different approaches to communicating oral health messages, in addition to quantitative feedback [5]. Saunders et al [6] recently described the use of free-text responses to evaluate the viewer’s acquisition of information from video clips. Rather than scoring text passages manually, using heuristic marking or a rubric, this approach scores them automatically relative to a large body of normative responses. An objective measure of information acquisition such as this has a number of potential applications, including, in our research, quantifying the benefit of video enhancements for people with low vision. In the present study, we examine whether crowdsourcing is an effective way to collect the required normative dataset. We test whether crowdsourced responses have substantial content and whether the responses, as well as the participants giving the responses, are similar to those seen in a supervised lab setting.

Crowdsourcing, first named by Howe [7], refers to the practice of advertising small self-contained tasks on the Web, usually to be worked on via the Internet, such as within a Web browser. Workers are typically compensated on the basis of the work they complete, rather than by a contract for a fixed amount of work. For the employer, the absence of the traditional relationship with employees, in many cases not knowing their identities or qualifications, is balanced by the speed and cheapness with which a large number of tasks can be completed. Often little time investment is required for data collection beyond the initial setup. The volume of data can compensate for potential inconsistency in quality: several studies have shown that combining the responses of nonexpert workers, whether by averaging or by using majority answers to screen out low-quality answers, can equal the quality of expert work, at a much lower cost [8-10]. The present study is based on the crowdsourcing website Mechanical Turk administered by Amazon, chosen because of its advertised worker base of over 500,000 individuals and because of the convenient infrastructure it provides for posting and paying for small jobs (typically requiring between 1 minute and 1 hour) to be completed over the Web. Because of the presence of an intermediary, workers can remain anonymous while receiving payments from experimenters.

Mechanical Turk and other crowdsourcing tools are particularly well suited for the task of collecting nonspecific normative datasets. Besides the speed and low cost of data collection, the population is relatively heterogenous, typically spanning a range of ages, educational backgrounds, and geographic locations that is greater than can be easily accessed by conventional methods [11,12]. Although it is difficult to control the demographic composition in crowdsourcing, this limitation is less serious when a general normative dataset sampling from the population at large is required. However, there is still concern about whether datasets collected in this way would have low quality responses or other distinctive characteristics that limit their usefulness. Some reasons why data obtained through recruiting and testing participants over the Internet might be less valuable [13,14] include potential sampling bias in recruiting only Internet users; that the experimental setting and display conditions cannot be controlled; greater chance of distractions and interruptions; and the possibility of worse compliance or motivation because of the lack of the presence of an experimenter. The question of the quality of more complex responses in light of these issues has not previously been addressed.

We compared a normative natural language dataset that was collected over the Web from participants recruited using Mechanical Turk (crowdsourced), with a dataset collected in the lab with participants recruited using conventional means (lab-sourced). As discussed in Saunders et al[6], the responses consisted of short descriptions of 30-second movie clips. We compared the results of the two recruiting processes, as well as the properties of the responses that the two groups produced. In addition to simple metrics such as the lengths of the responses, we used a take-one-out procedure to evaluate the quality of the content. The text of each response was compared to the text of all other responses in the same normative dataset, taking note of whether it was more similar to the responses to the same movie clip than to the responses to other movie clips, using a simple count of shared words. We also performed this procedure crossing the two normative datasets to test whether the content was similar.

## Methods

### Recruitment

Crowdsourced participants (workers) were recruited through postings on Amazon Mechanical Turk and were limited to workers who were registered as living in the United States. Demographic information was requested from each worker before they completed any tasks. At the end of the demographic survey, workers were informed about what they would be asked to do and actively consented by selecting a check box. Workers were known to us only by an ID assigned by Amazon. They were paid on a per-response basis, with Amazon as an intermediary. Workers were paid US$0.25 per response contributed, with a one-time $0.25 bonus for filling out the demographic survey and a $0.25 bonus for every 25 responses contributed and approved.

Lab-sourced participants were recruited from the community in and near Boston, Massachusetts, using a contact list or by being referred by participants in this and other studies. There was a target number of 60 participants divided equally into three age groups: under 60 years, 60-70 years, and greater than 70 years, each with equal numbers of men and women. The age stratification ensured responses from older participants, to investigate a possible age effect and because the visual disorders addressed in our other research, such as macular degeneration, are more prevalent in older people. Other criteria for the lab-sourced participants included a normal appearance of retina, no ocular conditions in self-reported ophthalmologic history, binocular visual acuity better than 20/32, and a Montreal Cognitive Assessment [15] score of at least 20. Informed consent was obtained from each participant prior to data collection, and they received a vision assessment and a cognitive assessment. Participants were shown the clips wearing habitual, not necessarily optimal, optical correction. They were compensated at a rate of $10 per hour, including the time for the clinical assessment, with the average time taken being approximately 2.5 hours.

### Video Clips

There were 200 video clips selected from 39 different films and TV programs, chosen to represent a range of genres and types of depicted activities. The genres included nature documentaries (eg, BBC’s Deep Blue), cartoons (eg, Shrek Forever After), and dramas (eg, The Hurt Locker). The clips were 30 seconds long and were selected from parts of the films that had relatively few scene cuts, which was reflected in the average number of cuts per minute in our clips being 9, as compared to approximately 12 per minute in contemporary films [16]. The clips included conversation, indoor and outdoor scenes, action sequences, and wordless scenes where the relevant content was primarily the facial expressions and body language of one or more actors. Although all participants heard audio in addition to viewing video, they were instructed to report only on the visual aspects of the clip.

### Data Collection

#### Crowdsourced Participants

**Figure 1:**
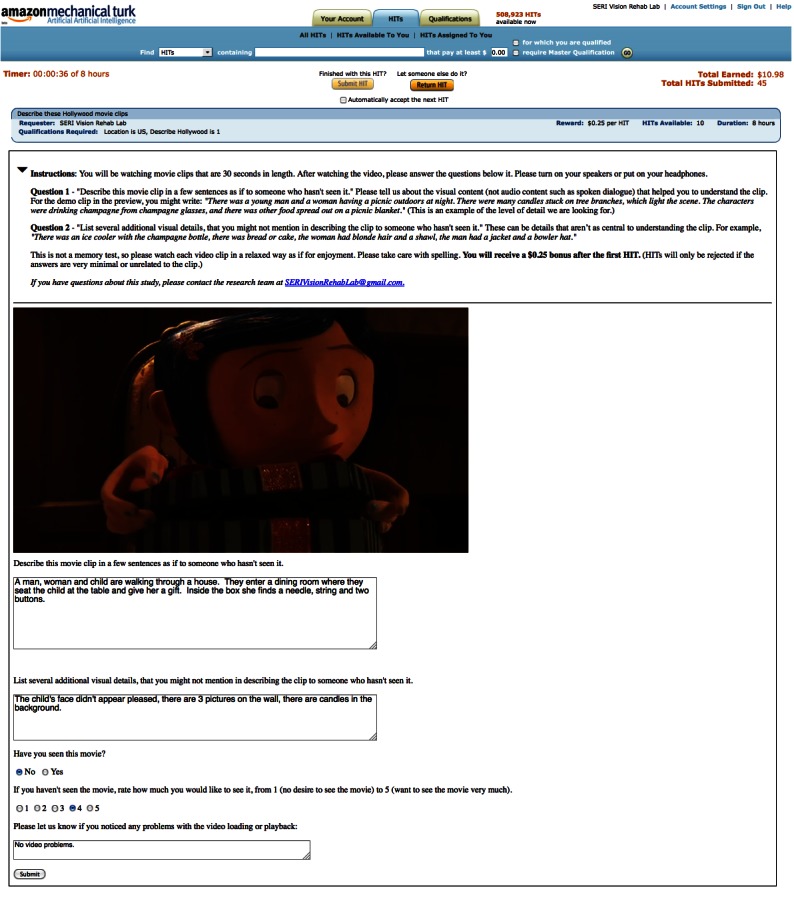
Example screenshot of Web forms used for data collection from crowdsourced participants, as hosted by Amazon Mechanical Turk.

Crowdsourced participants viewed the video clips within a Web browser, on a local computer of their choice. Therefore the size of the monitor, their distance from the monitor, and other display characteristics were not fixed. The clips were shown within the frame of the Mechanical Turk interface (Figure 1), with each clip representing a separate HIT (Human Interface Task, the unit of paid work on the Mechanical Turk website). Below the clip, there were two text boxes in which to answer two movie description prompts, “Describe this movie clip in a few sentences as if to someone who hasn’t seen it” and “List several additional visual details that you might not mention in describing the clip to someone who hasn’t seen it.” Text entry into these boxes was disabled until the clip had finished playing. Workers could complete as many video clip description tasks as they wanted while more clips were available, at any time of day. It was not possible to guarantee that each worker would complete a certain number of these tasks. The $0.25 bonus for every 25 responses was included as an inducement to complete more clips. Workers were prevented from seeing any clip more than once. Across all crowdsourced participants, 20 responses were collected for each clip, for a total of 4000 responses.

#### Lab-Sourced Participants

Lab-sourced participants viewed the video clips on an iMac i7 at a fixed distance of 100 cm. The videos were 33 degrees of visual angle wide. The clips were displayed by a MATLAB program using the Psychophysics Toolbox [17]. An experimenter gave the instructions and was in the room during data collection, but the MATLAB program automatically displayed the prompts after viewing a clip. The prompts were the same as for the crowdsourced participants. The spoken responses to each prompt were recorded using a headset microphone and later transcribed using MacSpeech Pro to produce the initial (automated) transcript, and then a separate group of Mechanical Turk workers verified and corrected the automated transcript [8]. Each lab-sourced participant viewed and responded to a different set of 40 clips selected from the set of 200 clips, for a total of 2400 responses (exactly 12 per clip). Equal numbers of responses to each video were collected for each of the six age-stratification by gender groups.

### Natural Language Processing

We processed the text of responses with the Text to Matrix Generator toolbox for MATLAB [18], which included a step that deleted a list of stopwords. Stopwords are words that carry little information on their own, such as “the” and “but”. To the default stopword list, we added verbal interjections that might have been transcribed from the lab-sourced verbal responses, such as “yeah” and “um”. The toolbox converted the compiled responses to term-document matrices for numerical analysis. We used the matrices to compute the number of words in responses and the relationship between demographics and number of words in responses. In addition, we evaluated the content by comparing responses to other responses that were made to the same video clip or to responses to other video clips. We reasoned that if a response contains accurate content about the clip, then on average it should be more similar to the responses to the same video clip than it is to responses to other video clips.

The method we used to compare responses was to count the number of words that two responses shared (after removing stopwords), disregarding repeated instances of the word in either response. More sophisticated approaches, for example that took into account synonyms, did not score as well in our validity benchmarks [6]. Since longer responses have an advantage as far as including words that might be found in normative dataset responses, the total number of words in a response (after removing stopwords) has a strong correlation with its shared word score, *r*=.63, across all the data we collected. However, the word count does not explain all the variance in the shared word score, and several composite scores, such as the ratio of shared words to total words, performed no better, so we used the simple shared word count as our metric of quality of responses.

This analysis was carried out within the lab-sourced and crowdsourced datasets. The similarity of the two response datasets was then evaluated by crossing the datasets: comparing responses from one dataset to the responses of the other dataset that originated from the same video clip. The mean of the resulting similarity scores should reflect the overall similarity in how the two populations described a clip. Finally, the two datasets were pooled and the mean shared words for each response, for the same clip and other clips, was computed relative to this pooled dataset.

## Results

### Participants

Data collection for the 60 lab-sourced participants (median age 64, range 23-85 years) required 13 months of active recruiting. Examination showed small subclinical cataracts in 6 participants: one case of red-green color vision deficiency, one case of dry eye, and one case of a detachment of the peripheral retina in the right eye, which had been repaired with laser surgery and should not have affected the ability to watch the video clips.

Data collection for the crowdsourced responses took place during 34 days of active data collection (over a 38-day period). Responses were contributed by 99 distinct Mechanical Turk worker IDs, which we assume corresponds to 99 individuals (median age 35, range 20-66 years). However, it is possible for a worker to create multiple accounts with the use of additional credit cards and email addresses (see Discussion). The number of responses contributed by crowdsourced participants ranged between 1 and 188, median 22, with the most prolific 20% of the workers contributing 60% of the responses. Responses were usually contributed over the course of multiple working sessions. The only eye condition reported by the crowdsourced workers that could have affected viewing was one case of cataracts (“not significant enough for surgery yet”). The same worker also reported having severe dry eyes. This worker contributed 39 responses.

The demographics of the two samples are presented in Table 1. The crowdsourced sample was skewed towards women, whereas equal numbers of men and women were recruited for the lab-sourced sample (by study design). The crowdsourced sample distribution had a younger median age, but a long tail of older workers (skewness=0.65). There was no evidence for a significant difference in the racial makeup of the two groups, although none of the lab-sourced sample reported their ethnicity as “Multiple”, in contrast to 8% of the crowdsourced sample. Similarly, there was not a significant difference in the number of people who reported themselves as “Hispanic”, although the proportion was higher in the crowdsourced sample. The lab-sourced sample was more highly educated, with a greater proportion of people with bachelor’s degrees and postgraduate degrees as their maximum attainment and a smaller proportion with a maximum attainment of “Associate degree” or “Some college”.

Table 1 also compares the demographics of the two samples to the population of the United States as a whole. The median age of the lab-sourced sample was older (by design), whereas the median age of the crowdsourced sample was 35 years, which is 2 years younger than the median age of the population of the country (2010 census [19]). Both samples resembled the United States in their racial and ethnic makeup to some degree, with the greatest discrepancy from the country as a whole being in fewer Asian people and fewer Hispanic-identified people. More people reported their race as “Multiple” in the crowdsourced population than in the United States as a whole. Both of our population samples had achieved a higher level of education on average than the population of the United States (based on people 18 years and over in the 2011 Current Population Survey[21]): there was a higher rate of bachelor’s degrees and a lower proportion who had attained only high school diplomas. This could have been partly due to the greater concentration of older adults in the two samples, with few participants falling in the 18-22 year range.

**Table 1:** Self-reported demographic characteristics of participants.

		Crowdsourced (N=99)	Lab-sourced (N=60)	Test for difference (*P* value)	US population
**Gender**				.14	
	Male	37 (37%)	30 (50%)		49% ^a^
	Female	62 (63%)	30 (50%)		51%
Age (median, min–max)		35y (20**–**66y)	64y (23**–**85y)	<.001	37y^a^
**Race/Ethnicity**				.18	
	Black	6 (6%)	5 (8%)		13% ^a^
	White	81 (82%)	54 (90%)		72%
	Asian	3 (3%)	1 (2%)		5%
	American Indian/Alaska Native	1 (1%)	0 (0%)		1%
	Multiple	8 (8%)	0 (0%)		3%
**Hispanic**				.09	
	Hispanic	8 (8%)	1 (2%)		16%^a^
	Not Hispanic	91 (92%)	59 (98%)		84%
**Highest education**				<.001	
	High school	11 (11%)	5 (8%)		35% ^b^
	Some college	16 (16%)	6 (10%)		23%
	Associate degree	32 (32%)	2 (3%)		10%
	Bachelor’s degree	28 (28%)	20 (33%)		21%
	Postgraduate degree	12 (12%)	27 (45%)		11%

^a^2010 United States Census [19,20].

^b^2011 US Current Population Survey, 18 years and over [21].

Finally, the self-reported demographics of our Mechanical Turk sample were similar to those found in a survey of Mechanical Turk workers taken in 2009 [22]. Like our workers, the workers in that study who were located in the United States had a mean age of approximately 35 years, were mostly women, and consisted of approximately 40% bachelor’s degree holders, with approximately 15% holding a postgraduate degree. Therefore, our sample represented a typical pool of American workers that are available for recruitment through Mechanical Turk for studies of this nature.

We compared survey answers about TV and movie viewing habits between the two sets of participants. We also asked about difficulties in viewing different display devices, with questions such as “Do you find it difficult to see details or feel that you miss important information when watching TV or movies on the TV?” A linear regression showed that crowdsourced participants watched more hours of TV, *t=*2.2, *P*=.03, with 38% reporting 3 or more hours a week compared to 25% in the lab-sourced sample, and 8% reporting 0-1 hours a week compared to 22% in the lab-sourced sample. Crowdsourced participants reported less difficulty with watching television, *X* ^2^_3_=10.5, *P*=.01, with 84% answering “never” or “rarely” to the difficulty question, compared to 73% of the lab-sourced participants. Far more crowdsourced participants reported having watched TV or movies on portable devices, such as a smartphone, than lab-sourced participants: 50% compared to 17%, *X* ^2^_2_=18.2, *P*<.001. However, for those individuals who did view media on portable devices, the level of difficulty reported was not significantly different between the groups, *X* ^2^_3_=1.5, *P*=.67. There was weak evidence of crowdsourced participants watching movies in the theater more often, *X* ^2^_6_=11.5, *P*=.07, although only 3% reported watching movies “never”, compared to 15% of the lab-sourced participants. There was no significant difference in the reported difficulty of watching movies, *X* ^2^_3_=5.0, *P*=.17, with most crowdsourced and lab-sourced participants (85% and 90% respectively) reporting difficulties “never” or “rarely”.

### Comparison of Lab-sourced and Crowdsourced Responses

**Figure 2:**
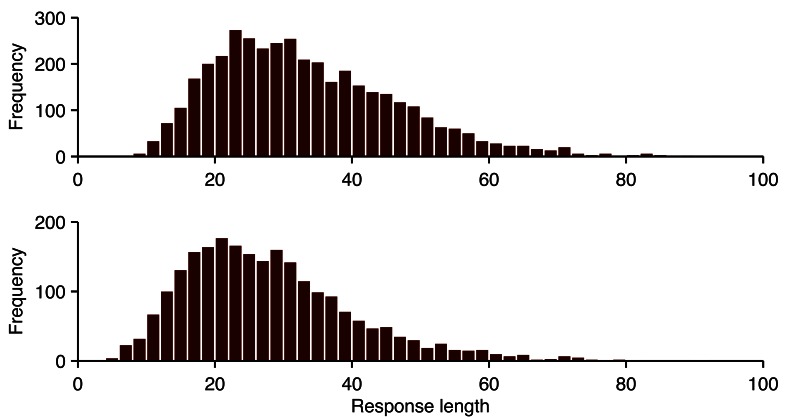
Distribution of number of words in responses in crowdsourced data (top) and lab-sourced data (bottom), after removal of stopwords.

The distribution of response lengths, after removing stopwords, between the lab-sourced and crowdsourced responses had a large overlap (Figure 2). The means were significantly different: *t*_6398_=15.1, *P*<.001, with the lab-sourced responses having 5 fewer words on average (mean 33 vs mean 28, medians 31 and 26). This difference could not fully be explained by differences in the demographics of the populations, as demonstrated by a mixed model analysis [23] with dataset, age, highest education level, and gender as predictors, and participant and video as fully crossed random factors. The lab-sourced dataset had shorter responses on average: *P*=.003, even when controlling for these demographic factors.

The total vocabulary used in the crowdsourced responses, after removal of words on the stoplist, was 8512 distinct words for 4000 responses, whereas the lab-source participants used 7356 words for 2400 responses. However, when we controlled for different dataset sizes, the lab-sourced participants had a more varied vocabulary. We randomly sampled 2400 responses from the crowdsourced responses, to match the lab-sourced dataset size, and computed the number of distinct words among those responses. From 1000 such samples, the average vocabulary size was 6904, compared to the lab-sourced vocabulary size of 7356, and this difference was significant: *t*_999_=319, *P*<.001. Using the same procedure to control for dataset size, we also compared the number of words that occurred once only in each dataset and found that there were 17% more in the lab-sourced dataset (3240 lab-sourced, 2770 crowdsourced), further supporting the idea that participants in the lab used a more varied vocabulary. The two complete datasets had 4875 words in common, with 3637 words appearing in the crowdsourced dataset but not the lab-sourced dataset, and 2481 words appearing in the lab-sourced dataset but not the crowdsourced dataset. Table 2 shows the most frequently used words in these two categories. Excluding stopwords, the mean word length was 4.1 letters for the crowdsourced data and 4.2 letters for the lab-sourced data.

**Figure 3:**
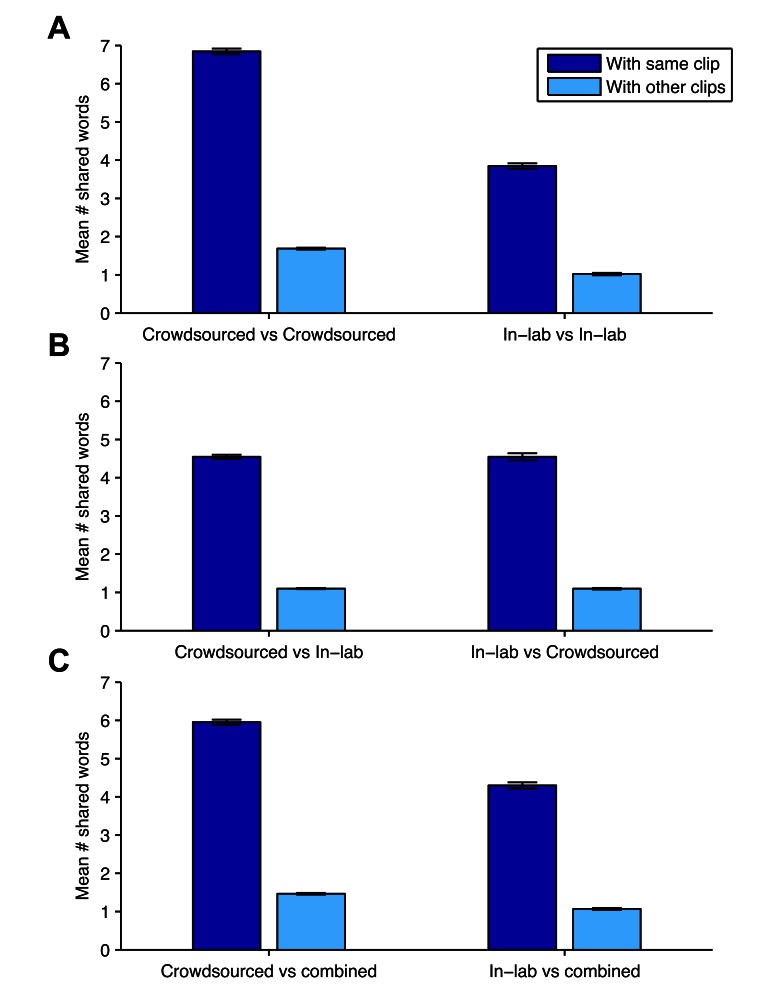
Mean number of words shared by responses with responses to the same clip, and with responses to other clips (error bars indicate 95% CI).

We used a take-one-out procedure to test the validity of the datasets and the scoring method. We compared each response to the remaining responses to the same clip, and to responses to other clips, using the same procedure of counting the non-repeating words shared between the two responses after removal of stopwords. In Figure 3, panel A illustrates that for both datasets, the average similarity to responses to the same movie clip was far greater than to responses to other movie clips: *F*_1,12796_=18,492, *P*<.001. There was also a difference due to the dataset, with the crowdsourced dataset having larger shared word scores on average than the lab-sourced dataset: *F*_1,12796_=3894, *P*<.001. There was an interaction between same/other comparisons and dataset, *F*_1,12796_=1580, *P*<.001, with the difference between the shared words with the same clip and other clips being larger in the lab-sourced condition, although the ratios between the same and other mean number of shared words were similar (4.0 in the crowdsourced dataset and 3.8 in the lab-sourced dataset).

We evaluated the similarity of the two datasets by performing the same response comparisons between datasets. Responses from the lab-sourced dataset were compared to the responses to the same movie clip in the crowdsourced dataset and to responses to other movie clips in the crowdsourced dataset. Panel B in Figure 3 demonstrates that the responses to the same clips were much more similar on average: *t*_4646.5_=120, *P*<.001 (Welch’s *t* test). Similarly, responses in the crowdsourced dataset were compared to responses to the same and other clips in the lab-sourced dataset, and the responses to the same clip were much more similar: *t*_2833.2_=71, *P*<.001 (Welch’s *t* test). Therefore we pooled the two datasets, and as panel C in Figure 3 shows, responses were much more similar to responses to the same clip than they were to responses to other clips, *F*_1,12796_=19263, *P*<.001. Additionally, crowdsourced responses had higher numbers of shared words on average, *F*_1,12796_=1363, *P*<.001, and a larger difference between same-clip and other-clip shared words, *F*_1,12796_=513, *P*<.001. The difference between crowdsourced and lab-sourced dataset shared word scores was not only due to demographic differences between the populations, as was shown by a mixed model with age, education, and gender as additional predictors, since the lab-sourced dataset still had significantly lower shared-word scores, *P*<.001.

Finally, we examined whether the average shared word score within a dataset for a particular clip (which reflects the homogeneity of responses to a clip) was similar between the two normative datasets. There was a significant correlation, *r*=.69, *P*<.001, between the mean of a video clip’s shared word scores in the crowdsourced dataset and in the lab-sourced dataset, indicating that clips that elicited a large amount of common vocabulary across respondents did so in both datasets.

### Demographic Effects

We conducted an analysis to determine whether age, gender, or maximum education level had an effect on average number of shared words within each normative dataset (that is, comparing responses to responses within the same dataset) or on the total number of words in responses (after removal of stopwords). We used mixed models with participant and video as fully crossed random factors. In the crowdsourced dataset, there was strong evidence that gender predicted shared word score, *P*=.004, with men having a shared word score that was 0.61 lower on average. Age was also a significant predictor of shared-word score, *P*=.01, with age positively related to shared-word score with coefficient=0.027 shared-words per year. Education level did not significantly predict shared-word score, *P*=.14. The relationship between demographic factors and total number of words approached significance for gender, *P*=.08; age, *P*=.07; and education, *P*=.06.

In the lab-sourced dataset, age predicted shared-word score, *P*=.01, but with a negative coefficient: –0.013 shared words per year. Gender and education did not significantly predict shared word score for the lab-sourced responses, ie, *P*=.53 and .24 respectively. Education significantly predicted the total number of words, *P*=.03, with a positive coefficient=2.3 words per education level, but gender and age did not: *P*=.38 and .11 respectively.

**Table 2:** Comparison of crowdsourced and lab-sourced vocabulary.

		Frequency
**Words that appeared in crowdsourced but not lab-sourced responses**		
	teen	36
	reveal	36
	raises	28
	cgi	28
	pony	23
	sleeves	22
	sheets	22
	listens	20
	maroon	19
	framed	18
	driveway	18
	storage	17
	whilst	16
	slicked	16
	rail	16
**Words that appeared in lab-sourced but not crowdsourced responses**		
	involves	15
	encampment	12
	drama	12
	beautifully	12
	recording	11
	movies	11
	jell-o	11
	report	10
	photographer	10
	fiction	10
	fella	10
	scenario	9
	involving	9
	impress	9
	disney	9

## Discussion

This study has shown that crowdsourced natural language data can have substantial content and be similar to data obtained in the laboratory. Although the demographic characteristics were somewhat different between the two samples, with the crowdsourced population being younger, less educated, and more female than the makeup of the lab-sourced population (which was selected for age and gender), there was a large overlap in the lengths of responses that participants provided, and in the vocabulary they used to describe specific movie clips. This makes crowdsourcing a feasible approach for collecting a large normative free-text dataset, such as is needed for an automated natural language scoring method [6]. Unlike previous applications of crowdsourcing to medical natural language processing (eg [24]), our method does not use worker qualification tests or “gold standard” responses created by experts to screen out low-quality answers. Instead, the large volume of free-text data compensates for potential inconsistency in the quality.

A consequence of this finding is that experiments using natural language responses can be conducted quickly using Web-based crowdsourcing, with the dependent measure being the scores obtained through automatic comparison to a normative dataset. These scores are taken to reflect the amount of valid content in the response. We are taking this approach to evaluate the benefits of image enhancement to acquiring information from video clips [25]. Furthermore, panel B in Figure 3 and the associated analysis demonstrate that a crowdsourced normative dataset can be used to effectively score free-text responses obtained in the lab. This makes it possible to use this approach to score a relatively small dataset obtained in a lab-based study, for example with a special population that would be hard to recruit online, against a large dataset of normative responses to the same query obtained through Mechanical Turk or a similar crowdsourcing service.

The crowdsourced sample resembled previous descriptions of the American Mechanical Turk population and resembled the population of the United States as a whole. The most discrepant feature of the crowdsourced population was the 2:1 gender imbalance towards female participants. This could help to account for the higher average shared word score in the crowdsourced sample because responses contributed by women received higher scores in general. Although the effect is relatively small, with average shared word score about 9% higher for women, it suggests that gender should always be included as a predictor in analyses of the scores produced by this method.

The crowdsourced participants also watched more television and movies and far more video on handheld devices. Since amount of TV watched, difficulties watching TV, frequency of watching movies, and likelihood of watching video on a handheld device have been found to be related to age [26], we conducted post hoc logistic and linear regressions that included age as a predictor. The difference in age distribution fully explained the difference in amount of handheld video watching and frequency of moviegoing. However, the difference in the number of hours of TV remained, so this was the only viewing habit difference between the populations that would remain if the samples were age-matched. There were only limited differences in the difficulty the two population samples reported in viewing video on different display devices, with both reporting the most difficulty with viewing on handheld devices and the least difficulty with viewing movies in the theater.

Besides the difference in the length of responses, the content of responses was more consistent in the crowdsourced dataset, indicated by the larger number of words shared between responses to the same clip. There were at least two major differences in the creation of the datasets that could have contributed: first, the crowdsourced responses were typed whereas the lab-sourced responses were spoken and then transcribed; and second, the lab-sourced population had a greater diversity, particularly in terms of age. Of the lab-sourced sample, 45% (27/60) was over 66 years, the age of the oldest crowdsourced participant. Examination of the words that appeared in one sample but not the other (Table 2) showed likely age-related vocabulary differences [27], such as “fella” in the lab-sourced sample and “cgi” in the crowdsourced sample. A more varied vocabulary in older participants would also explain the inverse relationship between age and shared word score in the lab-sourced dataset. However, as described in the results, a difference between the shared word scores of the datasets remained even when controlling for age. Whatever the cause, our results showed strong evidence for a more varied vocabulary in the lab-sourced dataset. However, despite this difference in word use, the shared word score differentiated within-clip responses from between-clip responses just as effectively when the lab-sourced normative dataset was used (panel A in Figure 3), so we saw no evidence that the scores, though lower on average, were less sensitive to semantic differences between the contents of clips.

Our comparison of crowdsourced and lab-sourced data collection focused on a specific type of natural language data, short descriptions of movie clips. One limitation is that, depending on the purpose of the data, responses may require different analysis techniques, which could increase the weight of the differences due to crowdsourcing that we found. For example, if responses are to be automatically scanned for a predefined list of keywords, then the increased probability of spelling errors when responses are typed could affect the results, as could the different vocabularies of the two populations. The fact that the two datasets differed both in their participant populations and in the manner of data input (typed or spoken) meant that differences could not be conclusively attributed to one or the other cause. However, our results show that neither difference led to a drastic change in the lengths or vocabulary of the responses. Another limitation is that we only report a simple method of scoring responses by counting the mean shared vocabulary with other responses to the same clip. Although this method had the best performance of the algorithms we evaluated [6], more sensitive methods of scoring responses might reveal more subtle differences between the datasets.

Data collection using Web-based crowdsourcing took only a fraction of the time it took us to recruit the target number of lab-sourced participants and was considerably less expensive, particularly when experimenter hours are taken into account. Including the time to identify, contact, and brief participants, we estimate an average of more than 3 hours per additional lab-sourced participant, compared to only a few minutes per additional crowdsourced participant. There is an investment of time and technical expertise to prepare a Mechanical Turk task, and data collection is not entirely automated, since it is necessary to review and approve submitted work and to answer worker queries [28]. There are also issues that arise that require time to resolve, such as one case we detected in a related study where an individual had set up two Mechanical Turk worker accounts (in violation of the website’s terms of service). Apart from this incident, we found little evidence of cheating, and along with the quantitative evidence of quality of responses, our observation was that the majority of the workers took a conscientious and thoughtful approach to answering our query. Although we had a mechanism for blocking particular workers from our jobs, we did not need to use it and had to reject only a tiny percentage of submissions (no more than 10 in the course of collecting 4000 responses).

There were several factors that may have contributed to our success with Mechanical Turk, given the conditions of the American worker pool at the time of data collection, August and September 2011. We were careful to stay in communication with the worker population, by offering an email address that we monitored closely and by introducing ourselves and responding to posts on the “turkernation” forums [28]. This helped to build our reputation as trustworthy employers and alerted us to problems and potential improvements in the Mechanical Turk task during the data collection period. Our task involved watching clips from entertaining films, which may have helped us attract more workers, led to more return visits, and helped to ensure engagement throughout the task. The free-text format of the response also allowed for a limited amount of creativity and self-expression. Other researchers [29,30] have noted the importance of fun in designing tasks for Internet users who have many competing options for how to spend their time, and this remains important even with financial incentives. However, we also had good results with a less stimulating task, correcting automated transcripts of the spoken lab-sourced responses. In both cases, each job was relatively short, approximately 2 minutes including the time for the clip to play, and we checked work and awarded payments frequently, typically every weekday. For the video description task, we offered a small bonus for every 25 responses contributed, but we did not see reliable evidence that this motivated workers to complete more tasks (ie, there were not noticeable spikes in the histogram of number of responses completed by workers just after 25, 50, or 75). We restricted the listings to workers registered in the United States, which some investigators have suggested might improve average submission quality[24], and we reasoned that focusing on predominantly English-speaking countries could be particularly helpful for natural language data collection (at the time of data collection it was only possible to restrict the task to workers from a single country).

Based on our experience, when might crowdsourcing not be a suitable replacement for lab-based data collection? Unlike in the lab-based data collection, we did not have control over the sample demographics. It might be possible to address this within Mechanical Turk, for example by rejecting workers who do not meet certain criteria [11], but this poses additional challenges because of lack of representation of some demographics in the pool of workers (in particular, older and lower income) [14] and in the difficulty of verifying self-reported demographic information. Our results suggest it would be difficult to obtain a target number of responses from all workers, which would be necessary for a balanced within-subject design (although see [31] for an example of this being achieved). However, the use of a mixed-model analysis can compensate for the lack of equal combinations of conditions and stimuli for each worker. Although we overcame most of the technical challenges inherent in presenting video clips to workers via the Web, the heterogeneity of hardware and software configurations meant that a few workers still experienced problems with the video playback, ranging from stuttering to a refusal to play. It was clear, too, that the exact color, contrast, and visual angle of the video would vary between participants, which was acceptable for a task evaluating high-level vision such as ours but could pose problems when low-level control of the stimuli is necessary. Nevertheless, we have demonstrated that it is feasible to crowdsource not only questionnaires or static images, but also multimedia. Finally, we observed that workers do not always read instructions carefully or else do not adhere to all the details consistently, which could make crowdsourcing unsuitable for experiments where manipulation of the instructions is a critical part of the experiment. As an example, our instructions stated that information from the soundtrack of the video clip should not be used in the description, but auditory information (including dialogue) was mentioned in a number of responses. To ensure the instructions are read and comprehended, a short quiz, as was recommended by Crump, McDonnell, and Gureckis [31], could be used.

In conclusion, crowdsourcing is an effective way to obtain natural language data quickly and inexpensively, both for collecting normative datasets and for conducting experiments. With respect to the concerns raised by the APA Board of Scientific Affairs [14], we found that using the crowdsourcing methodology we chose, the population sample resembled the population of the United States in several key demographic factors and that responses were of a high quality. Crowdsourcing can provide a valuable complement to more narrowly-targeted traditional recruiting and data collection methods and even substitute for them in some studies.
